# Identification of rice leaf disease based on DepMulti-Net

**DOI:** 10.3389/fpls.2025.1522487

**Published:** 2025-03-27

**Authors:** Kui Hu, Xinying Zheng, Xinyao Su, Lei Wu, Yongmin Liu, Zhenhua Deng

**Affiliations:** ^1^ School of Electronic Information and Physics, Central South University of Forestry and Technology, Changsha, Hunan, China; ^2^ Research Center of Smart Forestry Cloud, Central South University of Forestry and Technology, Changsha, Hunan, China; ^3^ School of Business, Hunan Normal University, Changsha, Hunan, China; ^4^ School of Bangor, Central South University of Forestry and Technology, Changsha, Hunan, China; ^5^ School of Forestry, Central South University of Forestry and Technology, Changsha, Hunan, China; ^6^ School of Automation, Central South University, Changsha, Hunan, China

**Keywords:** rice leaf diseases, convolutional Neural Network, DepMulti-Net, depthseparable convolution, multi-scale feature fusion, feature reuse

## Abstract

This research presents DepMulti-Net, a novel rice disease and pest identification model, designed to overcome the challenges of complex background interference, difficult disease feature extraction, and large model parameter volume in rice leaf disease identification. Initially, a comprehensive rice disease dataset comprising 20,000 images was meticulously constructed, covering four common types of rice diseases: bacterial leaf blight, rice blast, brown spot, and tungro disease. To enhance data diversity, various data augmentation techniques were applied. Subsequently, a novel VGG-block module was introduced. By leveraging depth-separable convolution, the model’s parameter quantity was significantly reduced. A multi-scale feature fusion module was also designed to effectively enhance the model’s ability to extract disease features from complex backgrounds. Moreover, the integration of the feature reuse mechanism and inverse bottleneck structure further improved the model’s recognition accuracy for fine-grained disease features. Experimental results show that the DepMulti-Net model has only 13.50M parameters and achieves an average accuracy of 98.56% in identifying the four types of rice diseases. This performance significantly outperforms existing rice leaf disease identification methods. In conclusion, this study offers an efficient and lightweight solution for crop disease identification, which holds great significance for promoting the development of smart agriculture.

## 1 Introduction

Rice is one of the world’s most crucial food crops, particularly in Asia, Africa, and Latin America, where it serves as the staple diet for over 50% of the population (Rashmi et al., 2025). The high yield and stable production of rice are vital for global food security. However, the frequent outbreaks of rice diseases severely impact its yield and quality, causing significant economic losses for farmers and posing a threat to global food security (Gagandeep et al., 2024). Common diseases such as rice blast, brown spot, and bacterial leaf blight can lead to substantial yield reductions if not detected and managed promptly (Chinna et al., 2024).

For instance, China has a vast rice - planting area of 29,450.1 thousand hectares, accounting for 24.89% of the country’s total sown area of food crops (Yuan, 2016). Approximately 60% of China’s population depends on rice as their staple food. The rice yield is of great significance for global food security. However, rice diseases occur frequently. In 2022, the area affected by rice diseases in China was around 0.62 billion hm² (Liu et al., 2023). Clearly, rice diseases can have a detrimental impact on yield, not only affecting farmers’ economic income but also directly threatening human food security (Yang et al., 2023).

Traditional disease - identification methods primarily depend on visual inspection and experience - based judgment by agricultural experts. These methods are not only time - consuming and labor - intensive but also subject to subjective biases, making it difficult to achieve rapid and accurate disease detection in large - scale farmlands (Teja et al., 2025). Moreover, with the increasing frequency and complexity of diseases resulting from global climate change and the intensification of agricultural activities, traditional methods can no longer meet the requirements of modern agriculture (Simhadri et al., 2024). In recent years, with the rapid development of artificial intelligence and deep - learning technologies, disease - identification methods based on computer vision have gradually become a research focus (Han et al., 2024). Deep - learning models, especially convolutional neural networks, have demonstrated excellent performance in image classification and feature extraction. They can automatically learn complex disease features from large amounts of image data, significantly improving the accuracy and efficiency of disease identification (Xu et al., 2022).

In the realm of rice disease identification, numerous researchers have made remarkable contributions. Chen et al. pre - trained the VGG network on the large - scale public dataset ImageNet to initialize the model’s weights. Subsequently, they transferred these pre - trained weights to a rice disease dataset for further training and achieved an average recognition accuracy of 92.00%. However, this method has a large number of parameters, which makes real - time identification challenging. Identifying rice leaf diseases in complex background environments remains a difficult task (Chen et al., 2020). Mannepalli et al. proposed an innovative approach using the VGG16 convolutional neural network to diagnose bacterial leaf blight, leaf - black - smut, and brown spot in rice leaves. Based on VGG16, the model utilized small convolutional kernels to extract rich features for image classification, achieving a classification accuracy of 97.77% for the three diseases. Although the model has a relatively large number of parameters, it laid the foundation for subsequent network development (Mannepalli et al., 2024). To reduce the number of model parameters, Zhou et al. proposed an improved YOLOv4 - GhostNet method for rice disease identification. This method combined the YOLOv4 object - detection algorithm with the lightweight GhostNet network. It achieved an average accuracy of 79.38% with a model parameter size of 42.45M. However, there is still room for improvement in the model’s recognition accuracy (Zhou and Liu, 2022). Weiwei Gao et al. proposed the YOLO V5 - EFFICIENT model based on YOLO V5s. They optimized the anchor box design using an improved K - Means algorithm, incorporated the CBAM attention mechanism in the Neck layer, replaced the BottleNeck Block with the RepVGG Block, and upgraded the SPPF module to S - SPPF, forming the YOLO V5 - EFFICIENT model. The mAP of the improved YOLO V5 - EFFICIENT model reached 89.2% (Weiwei Gao et al., 2024). Amitabha Chakrabarty et al. proposed an interpretable fusion model integrating lightweight CNN and transformer architectures for rice leaf disease identification. The model achieved a precision of 0.97, a recall of 0.96, and an F1 - score of 0.97. However, in complex background environments, the recognition accuracy of this method drops significantly (Chakrabarty et al., 2024).

In the field of other crop disease identification, many researchers have also made significant achievements. Amreen Abbas et al. generated synthetic tomato leaf disease samples using a generative network and then trained a DenseNet121 model via transfer learning. The classification accuracy for five types of tomato leaf disease images reached 97.11% (Amreen et al., 2021). Bracino et al. focused on apple leaf disease identification and extracted and selected features based on the color and texture of apple leaf diseases. However, multiple image - processing steps may lead to the loss of detailed information, thus affecting the classification accuracy (Bracino et al., 2020). Zhang et al. proposed a recognition model based on an improved ResNet - 50 architecture for apple leaf pests and diseases. The model integrated a Coordinate Attention (CA) module and Weighted Adaptive Multi - scale Feature Fusion (WAMFF) to enhance the image feature - extraction ability of ResNet - 50. Nevertheless, these methods have poor transferability and cannot be directly applied to rice leaf disease identification (Zhang et al., 2023).

Despite the significant progress in disease identification, three key issues remain unresolved: the complex background problem. Image data collected in laboratory environments, free from complex background interference, can achieve high recognition accuracy. However, rice leaf images captured in natural environments are easily influenced by complex backgrounds, resulting in a sharp decline in recognition accuracy (Haikal et al., 2024). Fine - grained identification of rice leaf diseases. Rice leaf lesions of different sizes can cause a reduction in recognition accuracy during feature extraction and classification. Some tiny lesions are easily overlooked (Zhou et al., 2024). The large number of parameters and complex structure of deep - learning models themselves require a large amount of rice leaf disease image training data. This makes them prone to overfitting and difficult to perform real - time identification (Yang et al., 2023).

The main objective of our research is to address the above - mentioned issues. We propose an economical and efficient method for rice leaf disease identification. The main contributions of this study are summarized as follows:

1. To improve the accuracy of rice leaf disease identification in complex backgrounds, we created an original dataset consisting of 5,000 rice disease images. These images were all captured in real - world paddy fields, differentiating them from laboratory - environment image data.
2. To enhance the feature - extraction ability for lesions of different sizes in complex backgrounds, we employed multi - scale feature fusion and feature - reuse techniques. This increased the network’s ability to extract key disease features, thus improving the model’s recognition accuracy.
3. By constructing a depth - separable convolution module that integrates feature reuse and multi - scale feature fusion through depth - separable convolution, we reduced the number of model parameters and accelerated model convergence. This ultimately enabled high - performance classification of rice leaf diseases.

The rest of this paper is organized as follows. Section 2 details the dataset, computational environment, and methodology used in this study. Section 3 presents the experimental results and analysis of the proposed method. Section 4 concludes the paper and points out potential future research directions.

## 2 Materials and methods

### 2.1 Self-build dataset

#### 2.1.1 Data acquisition

In this study, rice disease images were captured using a Sony A7M3 camera in Hunan Province, a major rice - producing region in China. To ensure that the dataset comprehensively represented real - world conditions, all images were taken in actual paddy field environments under both strong - light and weak - light conditions.

The captured images were saved in JPG format and resized to 224×224 pixels. This standardization was essential for preparing the data for model training, ensuring uniformity of input format and thereby enhancing model performance.

The dataset was then divided into training and test sets at a ratio of 8:2. Prior to training and testing, the data were randomly shuffled. This randomization step was crucial for unbiased model evaluation, ensuring that the model was tested on a representative sample of the data.

Details of the dataset, including the number of samples in each category and the sample images under different lighting conditions, are presented in **Table 1**. Compared with the public dataset PlantVillage, all images in our dataset were captured in real - world paddy field environments, fully considering outdoor noise conditions. In contrast, the images in the PlantVillage dataset were taken under laboratory conditions with a uniform background, which limits their applicability in real - world scenarios.

**Table 1:** Original dataset.

Diseases	Numbers	Strong light conditions	Weak light conditions
Bacteriablight	1000	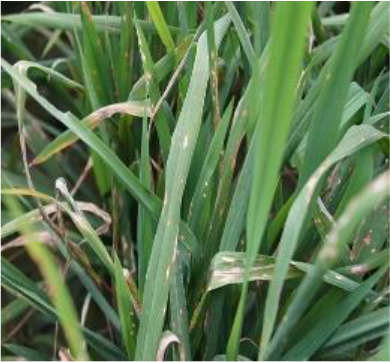	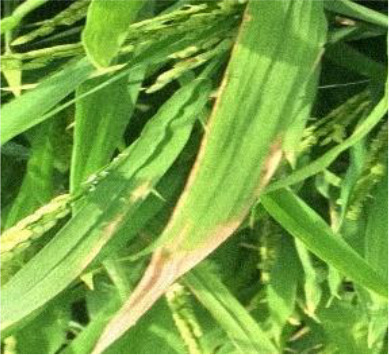
Blast	1000	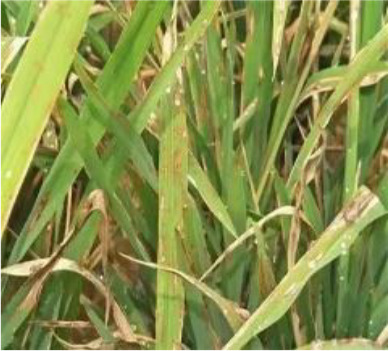	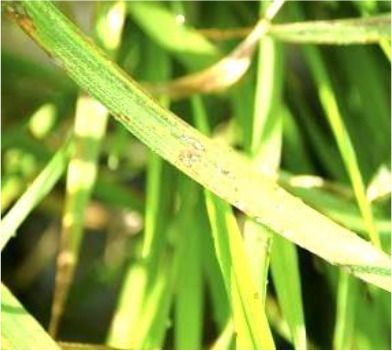
Brownspot	1000	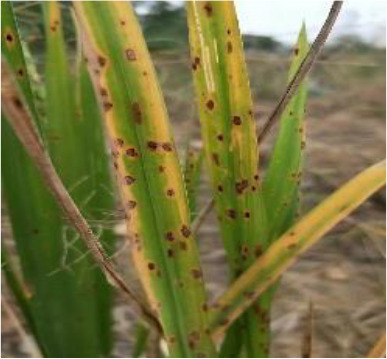	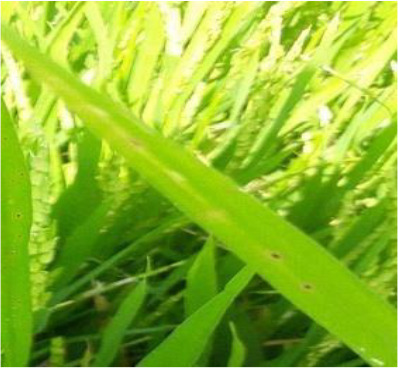
Tungro	1000	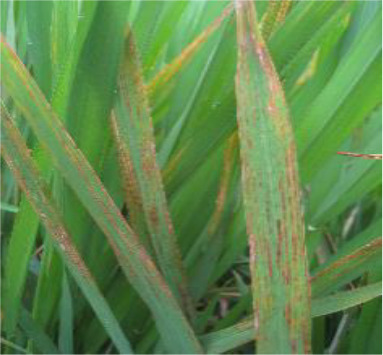	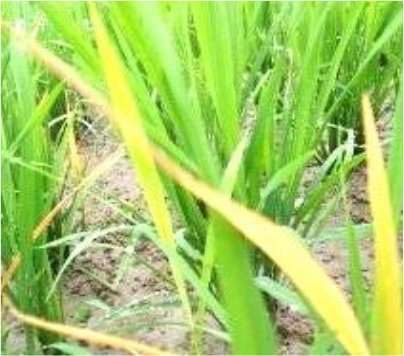

#### 2.1.2 Image enhancement of rice leaves

In actual real - world scenarios, such as in the middle of rice farmland, rice leaves are often exposed to strong direct light, interweave with each other, cast shadows on each other, and sway in the wind (Cheng et al., 2024). Strong direct light can affect the extraction of disease features by the model. The interweaving of rice leaves and the obstruction caused by overlapping leaves increase the difficulty of extracting disease features. Additionally, leaves that are blown or shaken by the wind can easily blur the image (Canbilen 2024).

**Figure 1:**
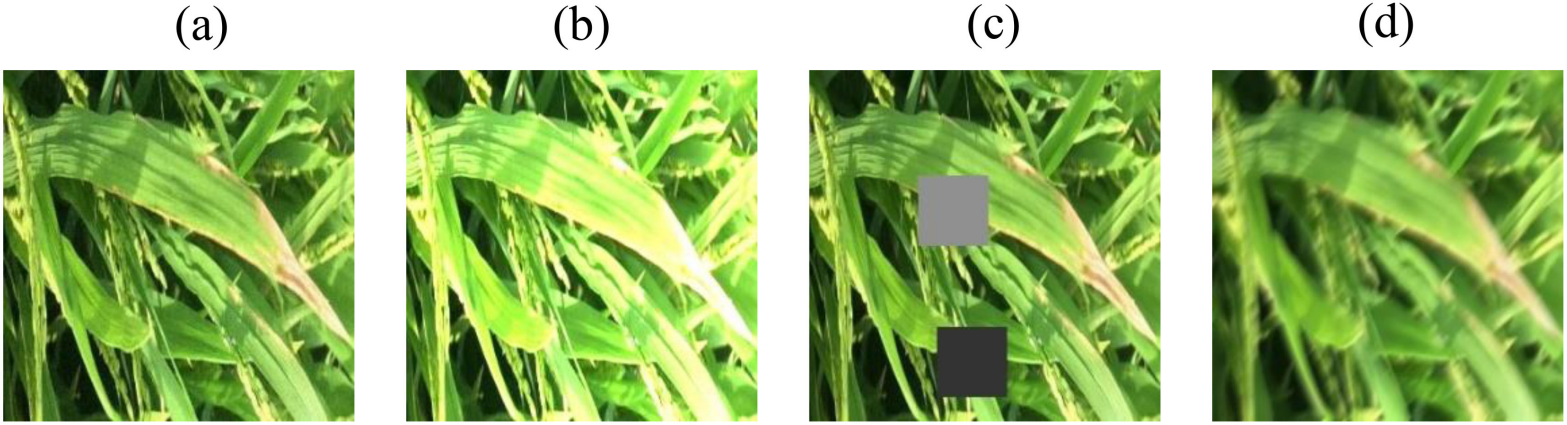
Sample images of pre-processing. **(a)** Gaussian noise **(b)** Random brightness **(c)** Random blocked **(d)** Motion blur.

Considering these practical factors, the present study employs image preprocessing methods such as Gaussian noise, random occlusion, random luminance, and motion blur (Too et al., 2019) to preprocess the original dataset. Each preprocessing method is applied to one original image. After batch preprocessing of the images in the original dataset, a new augmented dataset is created. During the training process, the model learns more disease features in complex environments, achieving the goal of simulating real - world scenarios and improving the accuracy of model validation. **Figure 1** shows some examples of image preprocessing.

Data augmentation methods are crucial in the field of crop pest and disease identification, especially for small or imbalanced datasets of pest and disease images. These methods aim to increase the sample size and balance the dataset, which is essential for deep - learning models with increasing depth and parameters (Lei et al., 2024). To address this issue, we used data augmentation techniques such as inversion, cropping, scaling, panning, and rotation to produce an enhanced dataset. This dataset contains four types of rice leaf disease images: 5,000 bacterial blight images, 5,000 blast images, 5,000 brown spot images, and 5,000 tungro images, for a total of 20,000 images. Specific statistics are shown in **Table 2**.

**Table 2:** Rice disease Aug-dataset.

Diseases	Total	Aug-dataset	Train-dataset	Test-dataset
Bacteriablight	1000	5000	4000	1000
Blast	1000	5000	4000	1000
Brownspot	1000	5000	4000	1000
Tungro	1000	5000	4000	1000
Total	5000	20000	16000	4000

### 2.2 DepMulti-Net model (depth separable VGG-inverted bottleneck multiscale feature multiplexing net)

**Figure 2:**
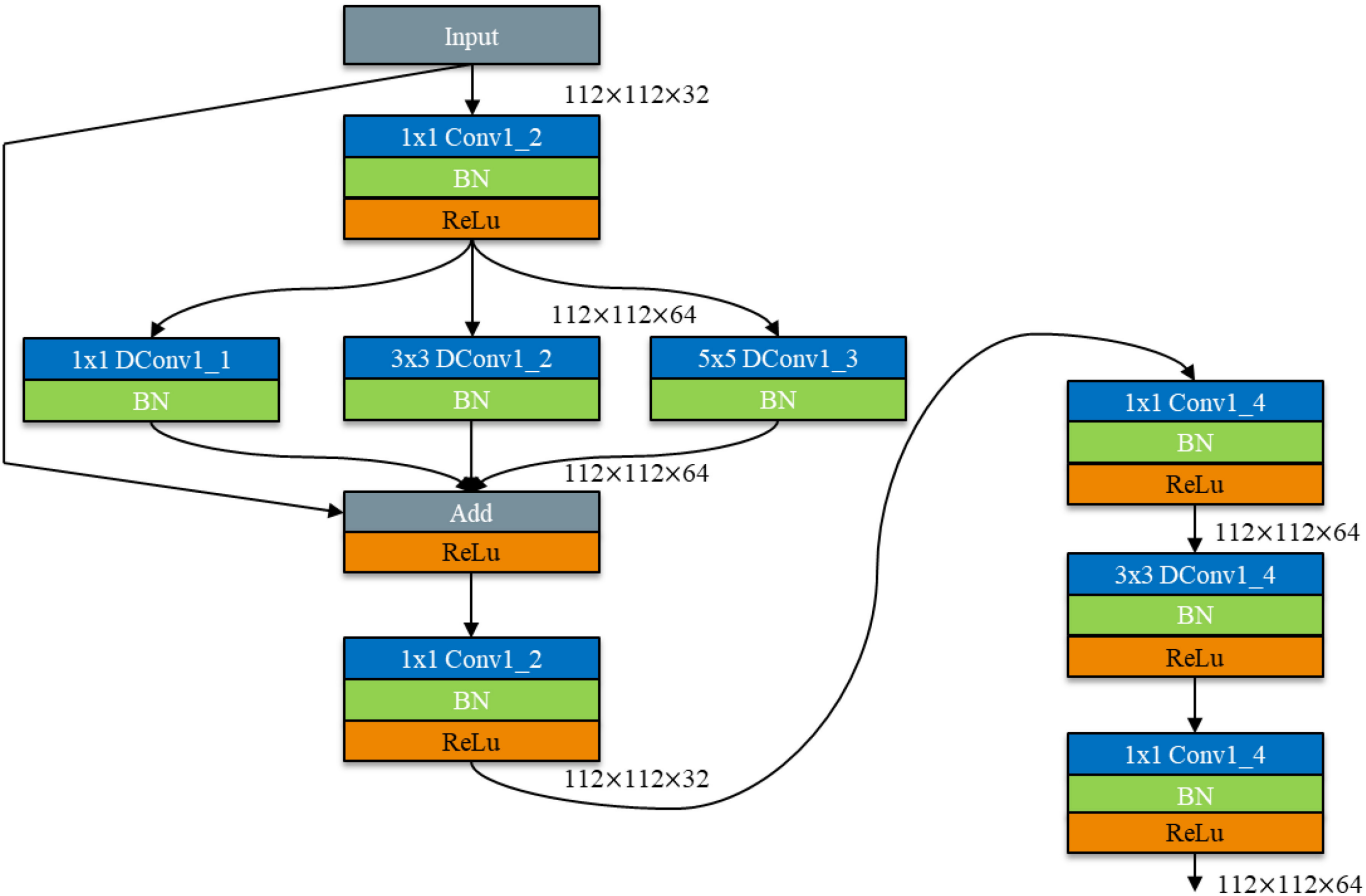
VGG - DepMulti-inverted bottleneck.

**Figure 3:**
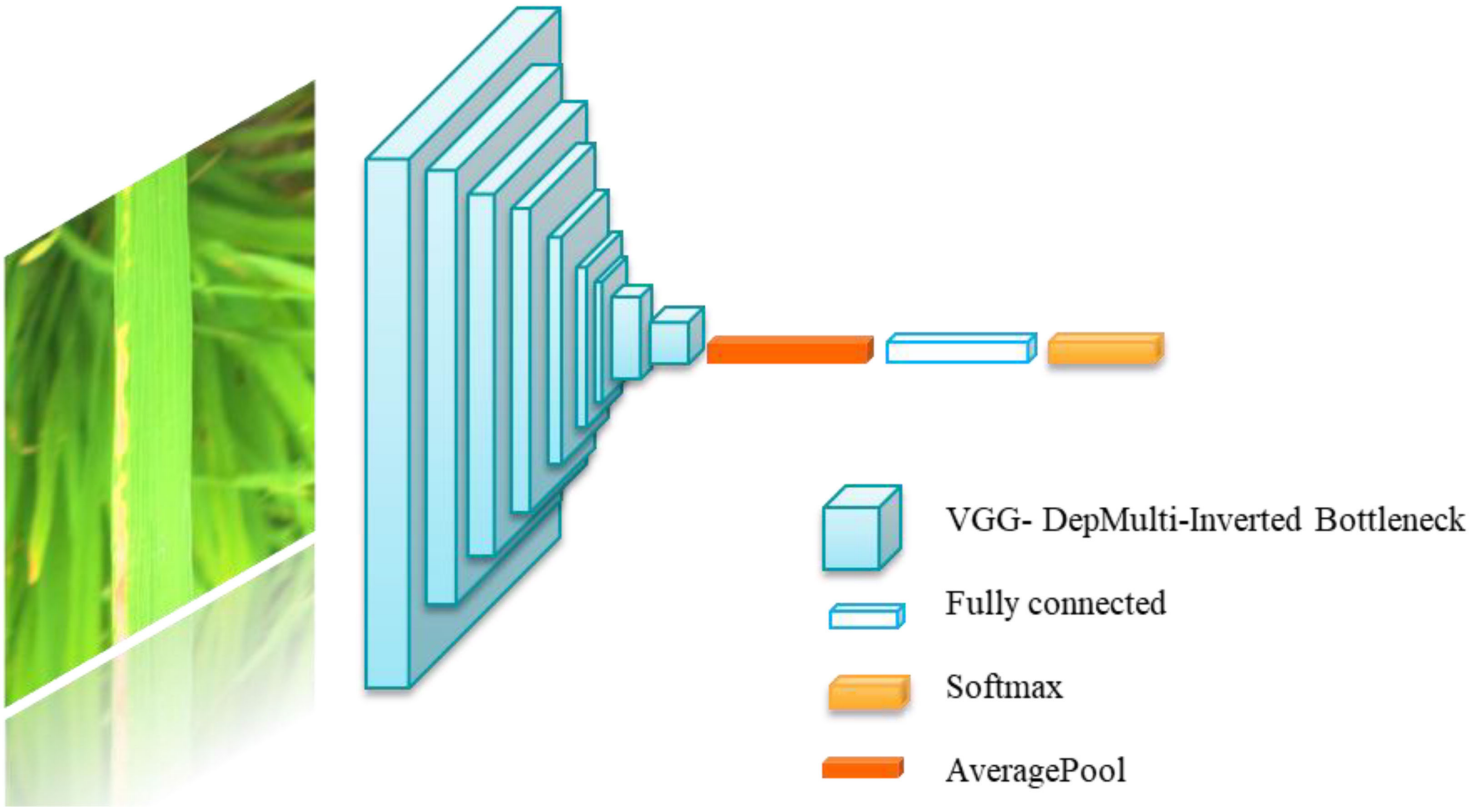
Overall process of DepMulti-Net rice disease recognition.

As shown in **Figures 2**, **3**, the model comprises nine DepMulti-Inverted Bottleneck modules, one mean pooling layer, and one fully connected layer. The input sample image size is 224×224×3. After feature extraction by the nine DepMulti-Inverted Bottleneck modules, the classification results are generated in the mean pooling layer and the fully connected layer, and are ultimately output via the classifier softmax.

#### 2.2.1 Depthwise separable convolution

Although convolutional neural network (CNN) models have demonstrated high accuracy in pest recognition, the substantial number of parameters presents several challenges. These include prolonged model training durations, the necessity for a large quantity of training samples, and difficulties in practical real - world applications (Lu et al., 2023).

**Figure 4:**
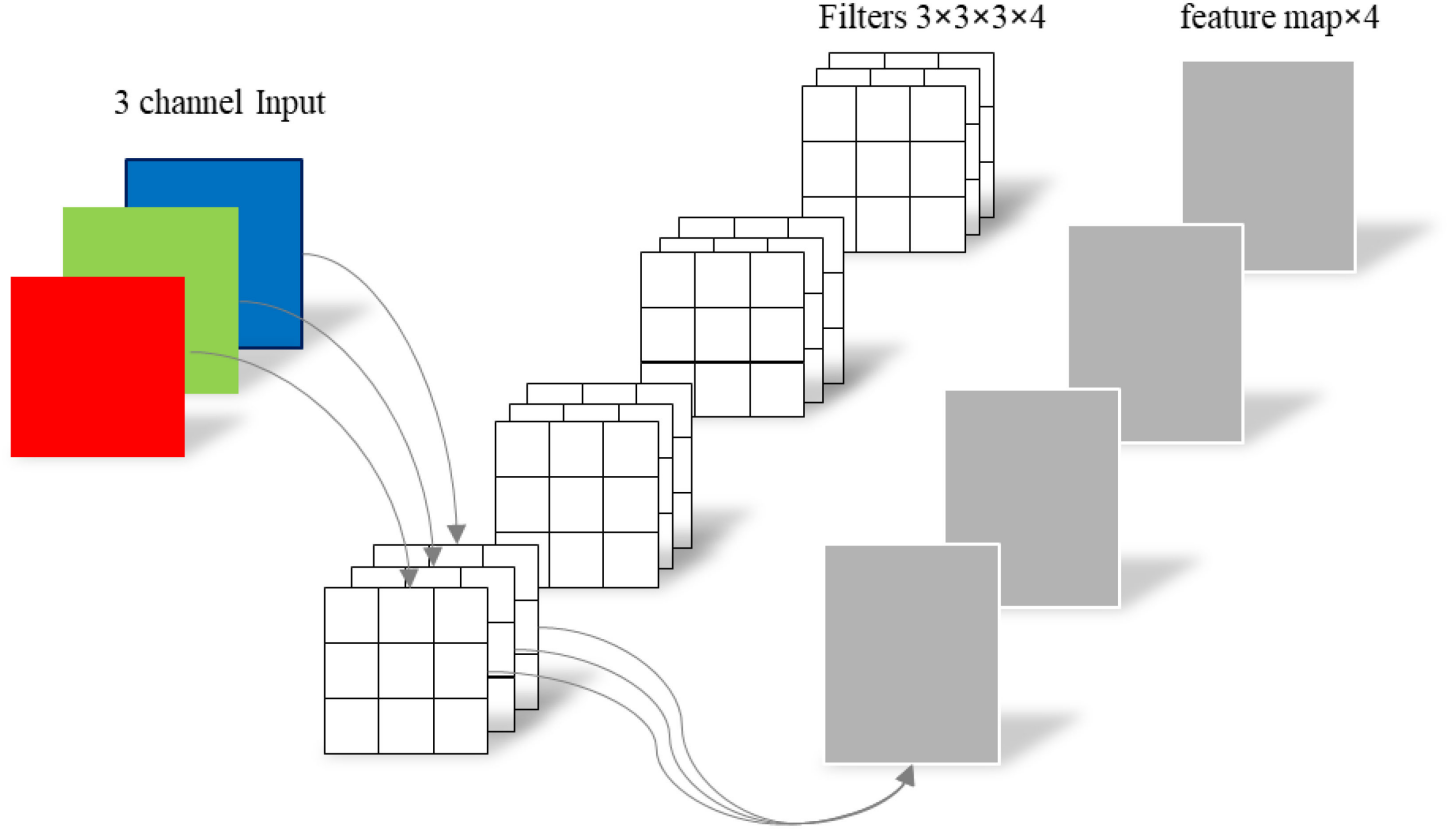
Standard convolutions.

**Figure 5:**
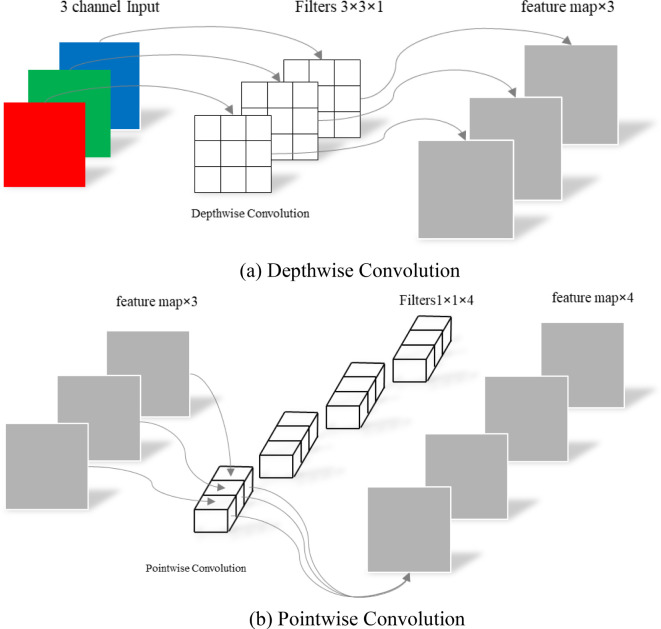
Depthwise separable convolution. **(a)** Depthwise Convolution. **(b)** Pointwise Convolution.

To address these challenges, the conventional standard convolution operation has been decomposed into channel - by - channel convolution (depthwise convolution) and point - by - point convolution (pointwise convolution). This decomposition can significantly reduce the number of parameters in CNNs. The differences between depth - separable convolution and traditional standard convolution are vividly illustrated in **Figures 4**, **5**.

In traditional standard convolution, a feature map is derived from the input 3 - channel RGB image. Each filter consists of three convolutional kernels, which independently perform convolution operations on each channel. This process yields three distinct convolutional feature maps. These feature maps are then combined through element - wise addition to generate the final output feature map. Essentially, each output feature map is the result of convolution operations performed on each input channel (Howard and Zhu, 2017).

In deep - learning models, each layer of the neural network generates a large number of feature maps, which correspondingly increases the volume of convolutional computations and the number of parameters. This computational complexity and the large number of parameters demand a substantial quantity of data for deep - learning models to learn effectively.

However, in practical applications, rice disease images are frequently scarce, and the availability of image samples is often limited. In the absence of a large number of data samples, deep - learning models are highly susceptible to overfitting, which can lead to a decrease in recognition accuracy. Additionally, the amount of computation and the number of parameters directly impact the speed of model recognition. Generally, the fewer the parameters, the more favorable it is for the practical application of the model in real - world testing scenarios. Thus, reducing the number of parameters in convolutional neural networks is a crucial challenge in the application - oriented research of deep learning.

Depth - separable convolution comprises two components: channel - by - channel convolution (depthwise convolution) and point - by - point convolution (pointwise convolution), as illustrated in **Figure 5**.

The first step involves performing channel - by - channel convolution on the input 3 - channel RGB image. In this process, the number of output channels matches the number of input channels, the number of filters equals the number of input channels, and each filter contains a single convolution kernel with a size of 3×3. As a result, the number of feature maps output through channel - by - channel convolution is equal to the number of input channels. The second step entails performing point - by - point convolution on the feature maps obtained from the channel - by - channel convolution. Channel - by - channel convolution is similar to standard convolution, but the convolution kernel size is uniformly 1×1. Compared with standard convolution, channel - by - channel convolution significantly reduces the number of parameters in the convolutional layer by decreasing the number of filters from 3 to 1 and the number of convolution kernels per filter from 3 to 1. This reduction in parameters is crucial for achieving a lightweight model design.

Channel - by - channel convolution performs separate convolution calculations for each channel, which means the interconnections between channels are lost. Therefore, point - by - point convolution essentially uses a 1×1 convolution kernel. This type of convolution does not consider the connections between pixels and their surrounding pixels; instead, it performs linear integration across channels. In other words, each feature map is linearly superimposed on the channels, thereby achieving dimensionality enhancement.

**Figure 6:**
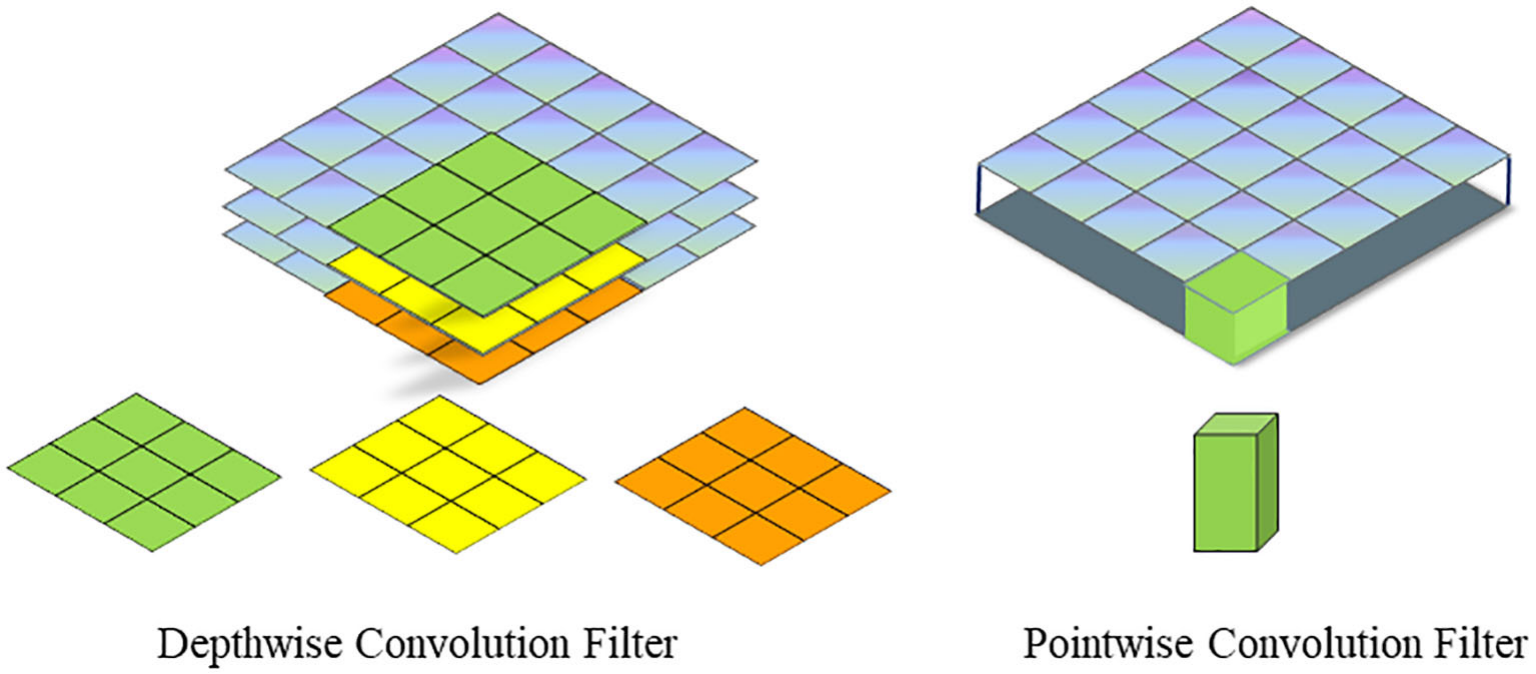
Spatial Schematic of Depthwise separable convolution.


The spatial schematic diagram of channel - by - channel convolution and point - by - point convolution is shown in **Figure 6**. The difference in parameter quantity between conventional standard convolution and depth - separable convolution is presented in Equation 1:

(1) $$\begin{array}{c} \frac{Parameters_{Depthwise\;separable\;convolution}}{Parameters_{Standard\;convolutions}}=\frac{D_{K}\times D_{K}\times M+M\times N}{D_{K}\times D_{K}\times M\times N}=\frac{1}{N}+\frac{1}{D_{K}^{2}} \end{array}$$

As an example, the convolution calculation shown in **Figures 4**, **5** can be obtained through Equation 2:


(2) $$\begin{array}{c} \frac{Parameters_{Depthwise\;separable\;convolution}}{Parameters_{Standard\;convolutions}}=\frac{3\times 3\times 3+3\times 4}{3\times 3\times 3\times 4}=\frac{39}{108}\approx \frac{1}{3} \end{array}$$

Based on the aforementioned formula, the number of parameters for depth - separable convolution can be reduced to (1/N+1/D_K_^2^) of that for standard convolution. For instance, considering the convolution calculations illustrated in **Figures 4**, **5**, with a convolution kernel size of 3×3, 3 input channels, and 4 output channels, the number of parameters for depth - separable convolution can be reduced to one - third of that for standard convolution.

#### 2.2.2 Depth-separable convolution-based VGG-block module

**Figure 7:**
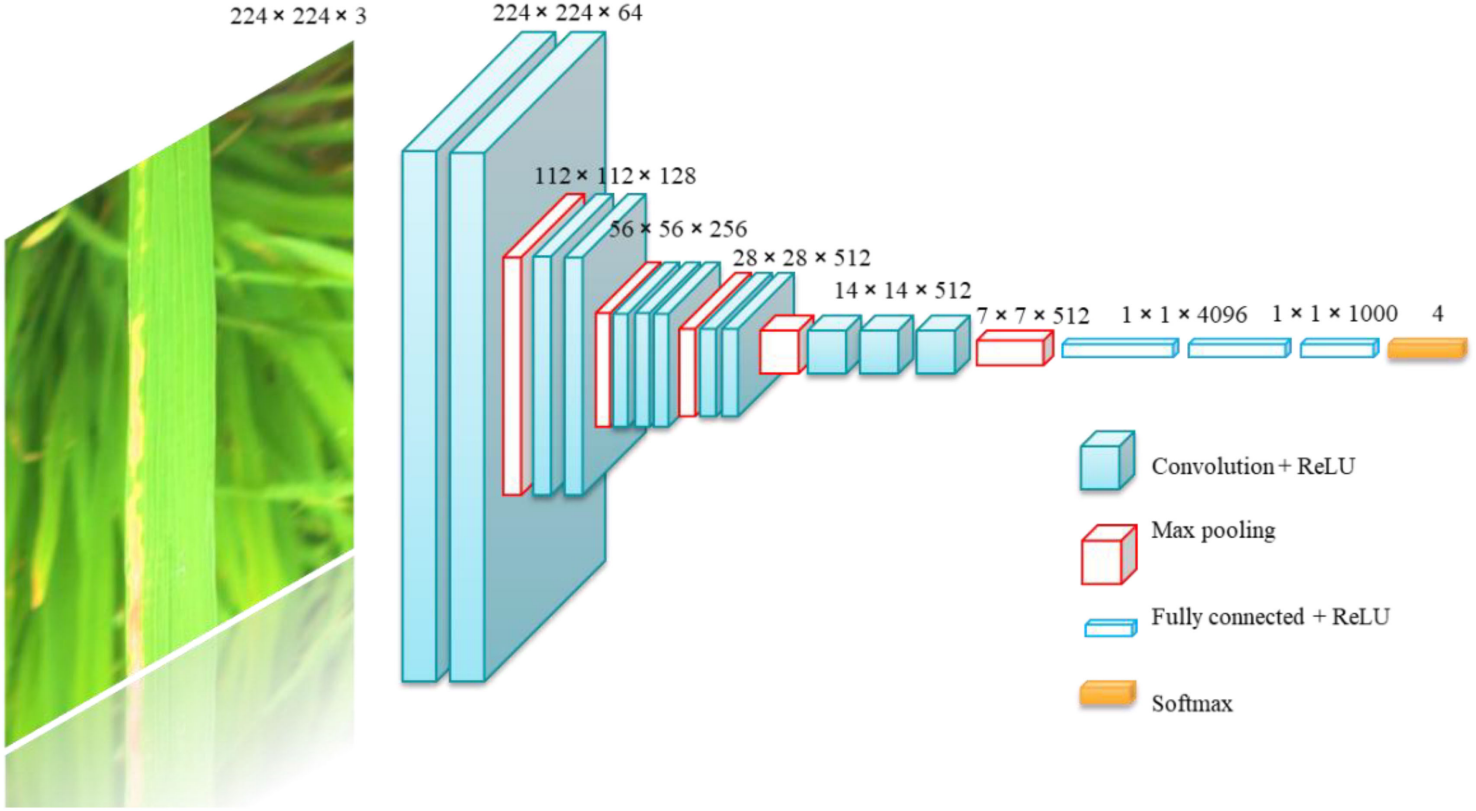
VGG16 Convolution neural.

In this study, VGG16 was employed as the baseline model. The VGG network streamlines the model construction process and increases the model depth through the use of convolutional blocks (Szegedy et al., 2014). The architecture of VGG16 is depicted in **Figure 7**.

The VGG16 network comprises 13 convolutional layers and 3 fully connected layers, with a model parameter count of approximately 138 million. The detailed parameters are presented in **Table 3**.

**Table 3:** Parameters of the VGG16 network.

Convolution layer	Kernel	Input Size	Channel	Params
Input	//	224×224	3	//
Conv1_1	3×3	224×224	64	1728
Conv1_2	3×3	224×224	64	36864
Pool 1	2×2	112×112	64	//
Conv2_1	3×3	112×112	128	73728
Conv2_2	3×3	112×112	128	147456
Pool 2	2×2	56×56	128	//
Conv3_1	3×3	56×56	256	294912
Conv3_2	3×3	56×56	256	589824
Conv3_3	3×3	56×56	256	598924
Pool 3	2×2	28×28	256	//
Conv4_1	3×3	28×28	512	1179648
Conv4_2	3×3	28×28	512	2359296
Conv4_3	3×3	28×28	512	2359296
Pool 4	2×2	14×14	512	//
Conv5_1	3×3	14×14	512	2359296
Conv5_2	3×3	14×14	512	2359296
Conv5_3	3×3	14×14	512	2359296
Pool 5	2×2	7×7	512	//
FC 1	//	1×1	4096	102760448
FC 2	/	1×1	4096	16777216
FC 3	//	1×1	1000	4096000
SoftMax	//	1×1	4	//
Total				138362328

**Figure 8:**
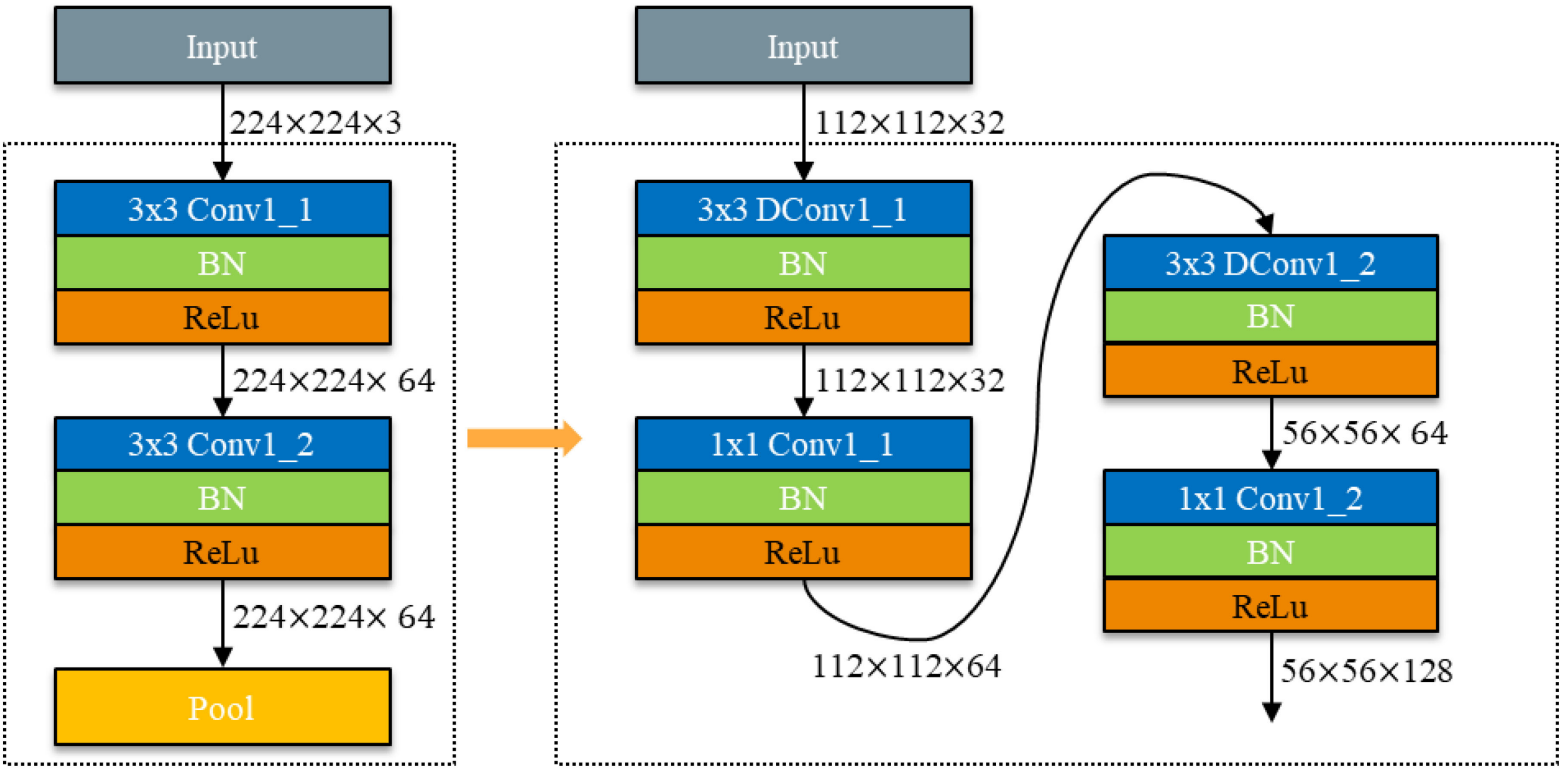
Schematic diagram of VGG-block conversion.

Given the substantial number of parameters in VGG networks, there is a pressing need to reduce the number of parameters in model lightweighting research. To this end, the VGG network was employed as a baseline for VGG block lightweighting research in this study. The VGG network comprises five VGG - blocks and three fully connected layers. Depth - separable convolution was utilized in place of the regular standard convolution in VGG - blocks. The conversion schematic is depicted in **Figure 8**.

The convolutional calculations in Equations 1 and 2 indicate that the number of parameters in a single VGG - block module is:


$${\text{Params}}_{\text{Conv}1\_1}=D_{K}\times D_{K}\times M\times N=3\times 3\times 3\times 64=1728$$

$${\text{Params}}_{\text{Conv}1\_2}=D_{K}\times D_{K}\times M\times N=3\times 3\times 64\times 64=36864$$

$${\text{Params}}_{\text{Conv}1}={\text{Params}}_{\text{Conv}1\_1}+{\text{Params}}_{\text{Conv}1\_2}=38592$$

The number of parameters for a single VGG - block module replaced by depth - separable convolution is:


$$\begin{array}{c} {\text{Params}}_{\text{DConv}1\_1}=D_{K}\times D_{K}\times M+M\times N\\ =3\times 3\times 32+32\times 64=2336 \end{array}$$

$$\begin{array}{c} {\text{Params}}_{\text{DConv}1\_2}=D_{K}\times D_{K}\times M+M\times N\\ =3\times 3\times 64+32\times 128=4672 \end{array}$$

$${\text{Params}}_{\text{DConv}1}={\text{Params}}_{\text{DConv}1\_1}+{\text{Params}}_{\text{DConv}1\_2}=7008$$

The above calculations demonstrate that replacing the standard convolution with depth - separable convolution reduces the number of parameters in a single VGG - block module to 18% of the original count. This number remains lower than the number of input channels, with a maximum of 32 channels.


$${\text{Params}}_{\text{DConv}1}/{\text{Params}}_{\text{Conv}1}=7008/38592\approx 0.18$$

Through the above theoretical derivation and mathematical calculations, it has been proven that using deeply separable convolution can greatly reduce the number of parameters in convolutional neural networks.

#### 2.2.3 Depth-separable convolutional module based on multiscale feature fusion

**Figure 9:**
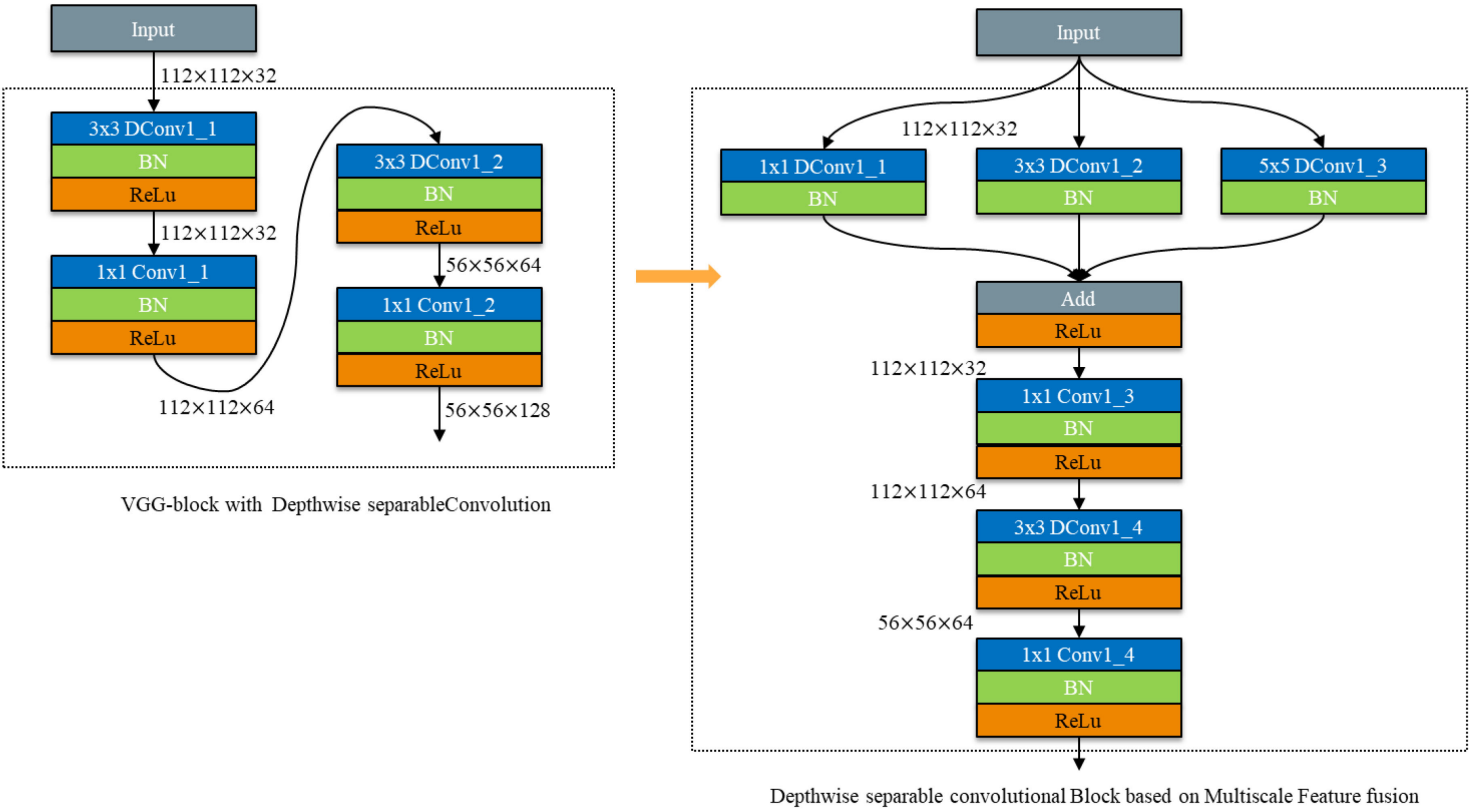
Depthwise separable convolutional Block based on Multiscale Feature fusion.

To address the challenges posed by complex background environments and the varying scales of disease spots, which can impact recognition accuracy, parallel convolutional kernel operators of different scales are employed. These operators are followed by feature fusion to effectively extract disease features corresponding to spots of different scales, thereby mitigating the influence of complex backgrounds (Shuli and Malrey, 2020). To ensure the accuracy of model recognition, a multi - scale feature fusion module was constructed. A depth - separable convolution module based on multi - scale feature fusion is proposed by cascading the depth - separable convolution module with the multi - scale feature fusion module. The structure is depicted in **Figure 9**.

Firstly, three different - scale feature extraction modules, with kernel sizes of 1×1, 3×3, and 5×5, are constructed using depth - separable convolution to extract disease features corresponding to spots of different scales. Subsequently, a depth - separable convolution module is cascaded. This approach aims to reduce the number of model parameters while improving the accuracy of model recognition.

#### 2.2.4 Depth-separable convolutional module based on feature multiplexing and multiscale feature fusion

While depth - separable convolution reduces the number of model parameters, it also decreases the number of convolutional kernels in each filter. This can lead to the loss of key disease features during feature extraction, resulting in lower recognition accuracy compared to conventional standard convolution. Therefore, balancing model recognition accuracy while reducing the number of model parameters is crucial for lightweight research.

To address this, a multi - scale feature fusion module is employed, followed by the cascading of residual connections from ResNet networks to deepen the model layers and enhance recognition accuracy (Liu et al., 2022). Residual connections in ResNet networks are a form of feature reuse, designed to mitigate the problems of gradient vanishing and network degradation. This concept has been widely cited in networks such as DenseNet and Transformer (Huang et al., 2017). Feature reuse not only addresses the issue of vanishing gradients but also prevents network degradation by allowing the model to bypass certain convolutional layers. If key features are lost in one segment of the convolutional layer, they can be re - extracted in the subsequent segment.

**Figure 10:**
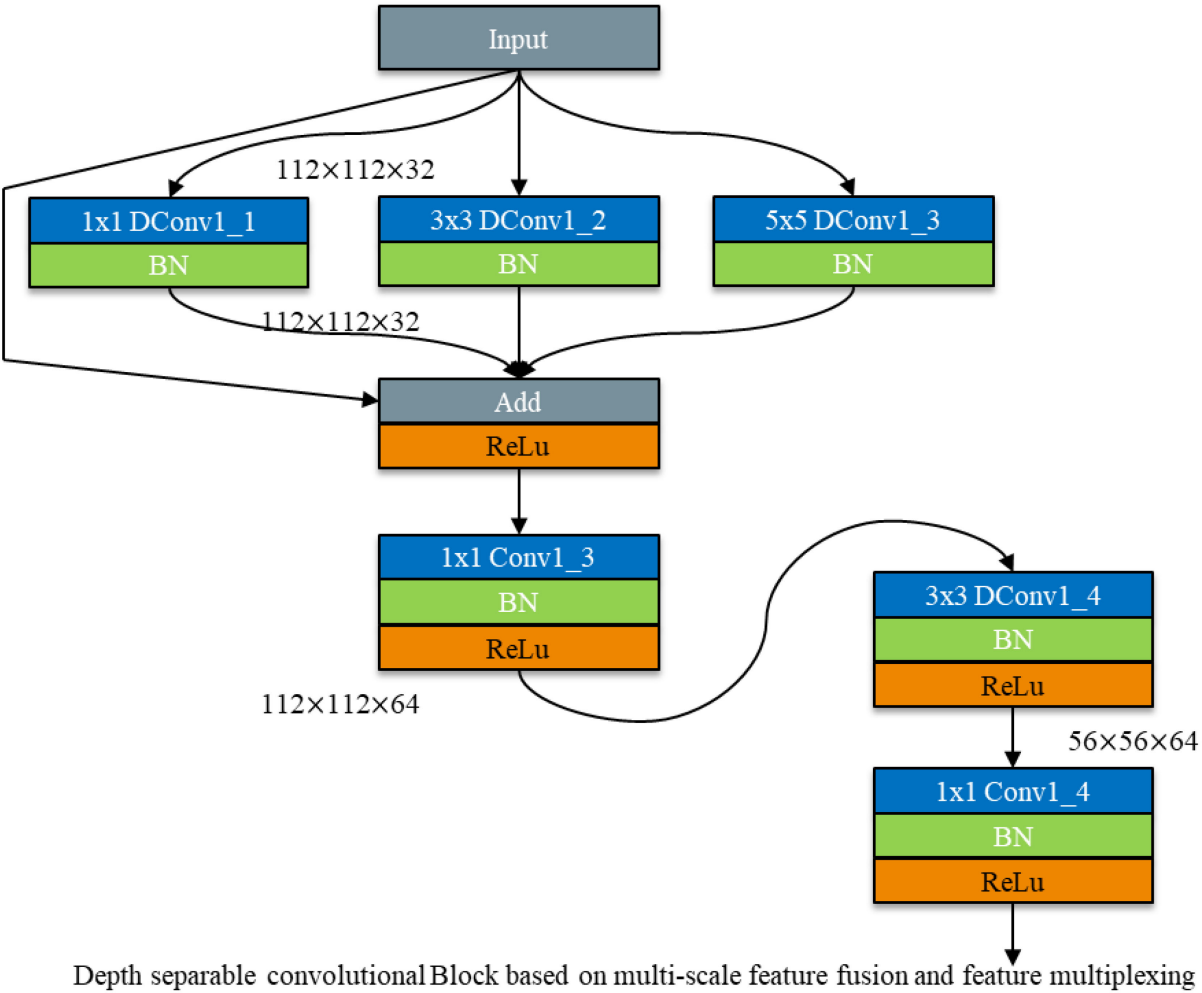
Depth separable convolutional Block for multi-scale feature fusion based on feature multiplexing.

Thus, this study integrates feature reuse into multi - scale feature fusion using depth - separable convolution to compensate for the accuracy loss caused by the reduction in convolutional kernels. The schematic diagram of the depth - separable convolution module for multi - scale feature fusion incorporating feature reuse is shown in **Figure 10**.

After employing the multi - scale feature fusion module, the features from the previous layer are reused. Feature reuse is achieved through Add feature fusion, where the feature maps are summed without altering the number of channels. This method increases the amount of information describing the features of an image while maintaining the dimensions of the image itself. The increased information per dimension is beneficial for the final classification of the image.

#### 2.2.5 Inverted bottleneck structure module based on feature multiplexing and multiscale feature fusion

**Figure 11:**
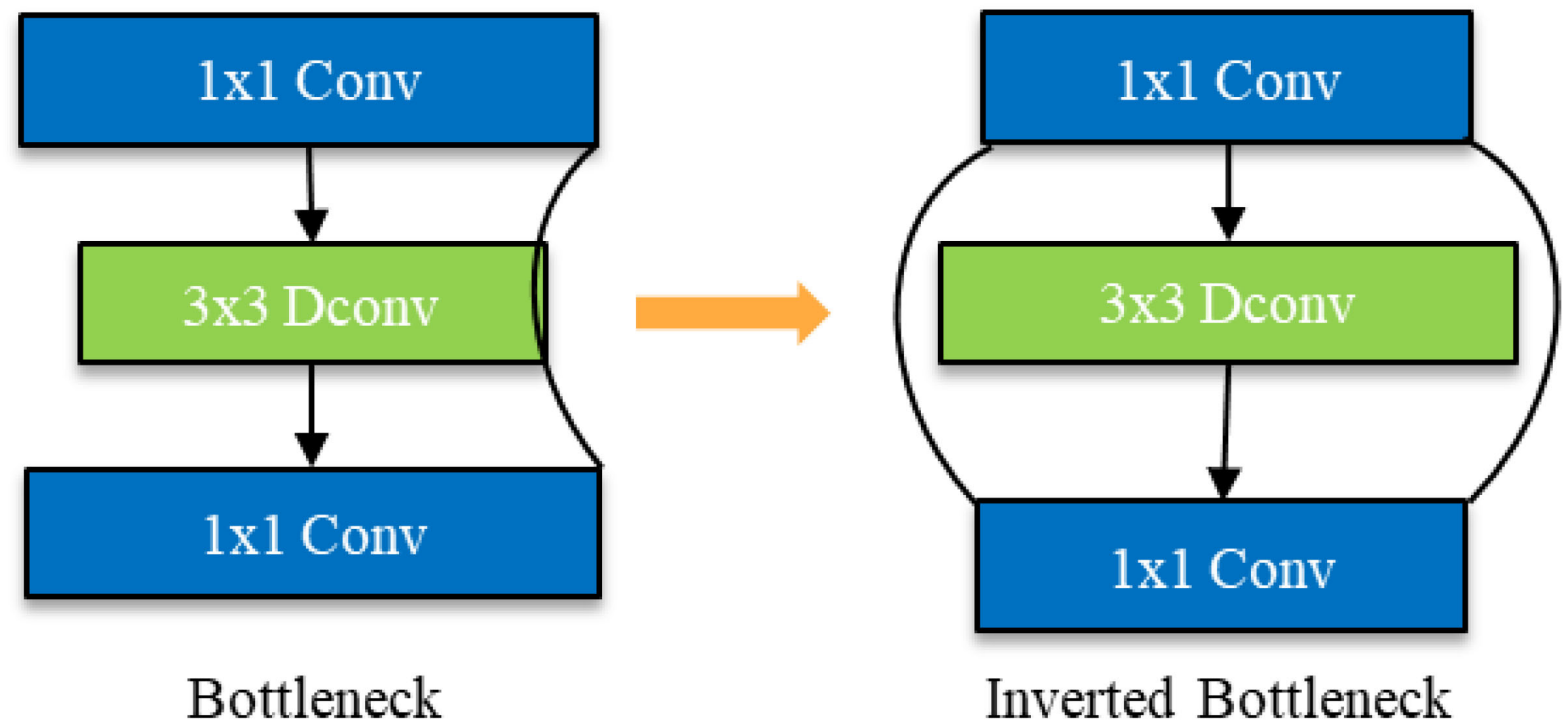
Schematic diagram of Bottleneck to Inverted Bottleneck.

Due to the reduction in the number of channels resulting from depth - separable convolution, the network is highly likely to lose a substantial quantity of feature information. To endow the network with the ability to extract more abundant feature information, we have transformed the commonly - used Bottleneck structure in convolutional neural networks into an Inverted Bottleneck structure, which has a spindle - shaped configuration. As shown in **Figure 11**, the Bottleneck is similar to that of a bottle, employing a dimensionality reduction mode before feature extraction. This approach has the advantage of reducing the number of channels and parameters, and is widely used in convolutional neural networks (Sandler et al., 2018).

However, after using depth - separable convolution, the convolution kernel is drastically reduced during the convolutional computation process. This leads to a reduction in the number of channels in the bottleneck structure and results in the loss of more feature information. Given the small scale of rice leaf disease features, the high similarity between different diseases, and the significant influence of natural environmental factors on images, disease feature extraction becomes particularly challenging. To more fully extract disease features, the bottleneck structure (Bottleneck) was changed to a spindle - shaped structure with an anti - bottleneck structure (Inverted Bottleneck).

**Figure 12:**
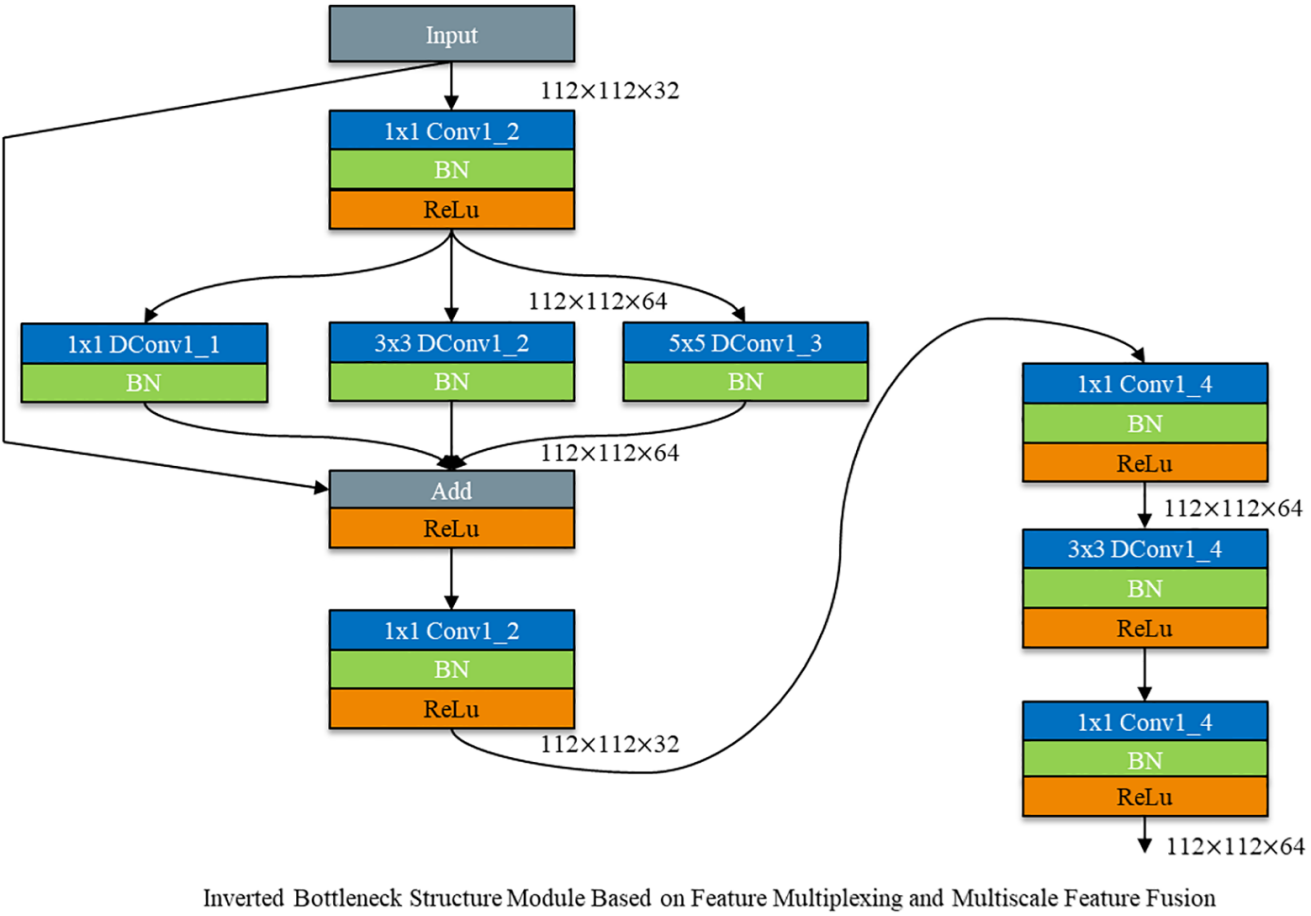
Schematic diagram of DepMulti-inverted bottleneck.

As shown in **Figure 12**, dimensionality ascension followed by convolution calculation is used to fully extract feature information. The spindle - shaped structure of the Inverted Bottleneck is fused with a depth - separable convolution module based on feature multiplexing and multi - scale feature fusion. This integration constructs a DepMulti - Inverted Bottleneck module based on feature reuse and multi - scale feature fusion. The schematic diagram of the module is depicted in **Figure 12**.

As shown in **Figure 12**, the DepMulti - Inverted Bottleneck module first performs a point - by - point convolution using a 1×1 convolution kernel to amplify the features of the input image. Subsequently, after normalization in the Batch Normalization (BN) layer and the introduction of nonlinearity via the ReLU activation function, convolution kernels of sizes 1×1, 3×3, and 5×5 are employed to extract features through channel - by - channel convolution. Finally, 1×1 point - by - point convolution kernels are used to reduce the dimensionality of the features. Feature reuse is then performed using the method of feature fusion via addition.

In the VGG16 network, the DepMulti - Inverted Bottleneck module replaces the VGG - block to reduce the number of model parameters. Meanwhile, the recognition accuracy of the model is enhanced by feature reuse methods and the spindle - shaped structure. To further reduce the number of model parameters, the three fully connected layers in the VGG16 network are modified to one mean pooling layer and one fully connected layer. The schematic diagram of the model structure is shown in **Figure 12**, and the specific computational parameters of the model are presented in **Table 3**.

After replacing VGG - blocks with DepMulti - Inverted Bottleneck blocks, the newly formed DepMulti - Net network comprises nine DepMulti - Inverted Bottleneck modules, as well as Conv2d, AvgPool, and fully connected layers. First, Conv2d is used to preprocess the input 224 × 224 × 3 image, resulting in a feature map of 112 × 112 × 32 with 864 parameters in this layer. Then nine DepMulti-Inverted Bottleneck modules are employed for feature extraction, with the number of parameters in each layer shown in **Table 4**. Finally, mean pooling is used to introduce nonlinearity, and four classification results are output after the fully connected layer.

**Table 4:** DepMulti-net model parameter.

Convolution layer	Kernel	InputSize	Channel	Params
Conv2d	3×3	224×224	3/32	864
DepMulti-Inverted Bottleneck	1×1, 3×3, 5×5	112×112	32/64	13056
DepMulti-Inverted Bottleneck	1×1, 3×3, 5×5	112×112	64/128	46592
DepMulti-Inverted Bottleneck	1×1, 3×3, 5×5	56×56	128/128	125952
DepMulti-Inverted Bottleneck	1×1, 3×3, 5×5	56×56	128/256	175104
DepMulti-Inverted Bottleneck	1×1, 3×3, 5×5	28×28	256/256	481280
DepMulti-Inverted Bottleneck	1×1, 3×3, 5×5	28×28	256/512	677888
DepMulti-Inverted Bottleneck	1×1, 3×3, 5×5	14×14	512/512	1880064
DepMulti-Inverted Bottleneck	1×1, 3×3, 5×5	14×14	512/1024	2666496
DepMulti-Inverted Bottleneck	1×1, 3×3, 5×5	7×7	1024/1024	7430144
AvgPool	7×7	7×7	1024/1024	//
FC	//	1×1	1024/4	4096
SoftMax	//	1×1	4	//
Total				13501536

Through the above modifications, the total number of parameters in the VGGNet network is reduced from 138 million to 13.50 million, and the number of parameters in the newly constructed DepMulti - Net network is only 9.7% of that in the VGGNet network.

### 2.3 DepMulti-Net model memory requirements analysis

The neural network model proposed in this study has a parameter count of 13,501,536. Typically, model parameters are stored in the form of 32-bit floating-point numbers, with each parameter occupying 4 bytes of memory space. Therefore, just the model parameters require approximately 54,006,144 bytes of memory, which is about 51.5 MB. However, when running the model, in addition to the storage of parameters, extra memory is needed to store intermediate activation values, which is usually 1-2 times the parameter counts. Nevertheless, when the model is deployed on agricultural drones or smartphones for inference, real-time gradient computation is usually not required, so this part of memory requirement can be neglected. Taking all factors into account, the actual memory requirement for this model during operation is around 100 MB. Modern smartphones typically have 4GB - 12GB of memory, while drones usually have memory ranging from several hundred MB to several GB, with high-end professional drones possibly equipped with 2GB - 4GB of memory. The memory requirement of this model is within its bearable range.

## 3 Experimental results and analyses

### 3.1 Experimental environment

The experimental software environment consists of a Windows 10 64 - bit operating system, the PyTorch deep - learning open - source framework, and Python as the programming language. The hardware configuration includes 16 GB of RAM, an AMD Ryzen 7 5800H processor with Radeon Graphics, and an NVIDIA GeForce RTX 3070 Laptop GPU, which accelerates image processing tasks.

### 3.2 Experimental settings

In this study, the SGD optimization algorithm was employed with the CrossEntropyLoss loss function. The training configuration included a batch size of 32, 20 training epochs, an initial learning rate of 0.01, and a momentum of 0.9.

### 3.3 Evaluation metrics

When training a classification recognition model, the input sample data are divided into four categories, corresponding to the four main variables defined in the confusion matrix: TP (True Positive) denotes the number of samples with positive true values and positive predicted values; TN (True Negative) denotes the number of samples with negative true values and negative predicted values; FP (False Positive) denotes the number of samples with negative true values and positive predicted values; and FN (False Negative) denotes the number of samples with positive true values and negative predicted values. Four main evaluation metrics were calculated to assess the performance of the classification model in deep learning tasks.

Accuracy: Accuracy is the proportion of samples with completely correct predictions to the total number of samples. It is typically used to evaluate the overall classification performance of deep learning models, including both positive and negative samples. The calculation formula is shown in Equation 3.


(3) $$\begin{array}{c} Accuracy=\frac{TP+TN}{TP+TN+FP+FN} \end{array}$$

Precision: Precision is the proportion of all predicted positive samples that are actually positive. It represents the accuracy of predicting positive samples and is calculated as shown in Equation 4.


(4) $$\begin{array}{c} Precision=\frac{TP}{TP+FP} \end{array}$$

Recall: Recall is the proportion of all samples with positive true values that are correctly predicted as positive. A higher recall rate indicates a higher probability of predicting true positive samples. The formula is shown in Equation 5.


(5) $$\begin{array}{c} Recall=\frac{TP}{TP+FN} \end{array}$$

F1 score: The F1 Score represents the harmonic mean of Precision and Recall, providing a balanced measure of the two. It is calculated as shown in Equation 6.


(6) $$\begin{array}{c} F1-score=\frac{2\times \text{Precision}\times \text{Recall}}{\text{Precision}+\text{Recall}} \end{array}$$

### 3.4 Ablation experiments

To verify the feasibility of the proposed method in this study, the depth - separable convolution module, multi - scale feature fusion module, feature reuse module, and inverted - bottleneck structure were tested and compared on the VGG network. Firstly, a comparative test of depth - separable convolution was conducted on VGG by replacing the standard convolution with depth - separable convolution to evaluate the model’s parameters, accuracy, and other performance indicators. Subsequently, based on these results, additional comparison tests were performed on multi - scale feature fusion, feature reuse, and the inverted - bottleneck structure. These three structures were integrated into the VGG network, and their combined effect on performance enhancement was compared to that of the original model.

As shown in **Tables 5**, **6**, the original VGG - 16 network achieved an accuracy of 95.79% with 138 million parameters. After replacing the standard convolution with depth - separable convolution, the model’s parameter count was reduced to 12.47 million, and the recognition time for a single image decreased from 0.296 seconds to 0.032 seconds. However, the network’s accuracy decreased by 3.02 percentage points. Although depth - separable convolution significantly reduced the number of model parameters, it also lowered the recognition accuracy. The reduction in the number of channels and convolution kernels can lead to the loss of disease feature information.

**Table 5:** Parameters rate accuracy of different approaches.

Model	Parameters/million	Rate/s	Accuracy rate
VGG-16	138.36M(138362328)	0.296s	95.79%
VGG- Depthwise separable convolution	12.47M(124700252)	0.032S	92.77%
VGG- multi-scale feature fusion	13.01M(130167905)	0.053s	96.41%
VGG-Feature Multiplexing	13.01M(130167905)	0.060S	97.53%
DepMulti -Inverted Bottleneck(DepMulti-Net)	13.50M(13501536)	0.073s	98.56%

**Table 6:** The recognition accuracy of different diseases for each method.

Model	Bacteriablight	Blast	Brownspot	Tungro
VGG-16	97.00%	95.33%	95.00%	94.80%
VGG- Depthwise separable convolution	91.67%	93.08%	92.86%	93.64%
VGG- multi-scale feature fusion	96.50%	96.33%	96.67%	96.4%
VGG-Feature Multiplexing	97.30%	97.67%	97.60%	97.40%
DepMulti -Inverted Bottleneck(DepMulti-Net)	98.625%	98.50%	98.60%	98.40%

After introducing the multi - scale feature fusion module, the number of model parameters increased from 12.47 million to 13.01 million, and the recognition accuracy improved to 96.41%, an increase of 3.64 percentage points. The recognition time increased by 0.021 seconds. After incorporating the feature reuse module and using the Add method to keep the number of model parameters unchanged, the network’s recognition accuracy improved to 97.53%, an increase of 1.12 percentage points. The recognition time for a single image increased to 0.060 seconds.

After introducing the inverted - bottleneck structure, the network’s recognition accuracy improved to 98.56%, an increase of 1.03 percentage points. Due to the additional convolutional layers, the number of parameters increased slightly by 0.13 million. However, compared with the original VGG - 16 network, the overall reduction in the number of parameters was 124.86 million, and the accuracy improved by 2.77 percentage points. The number of parameters in the improved model is only 9.7% of the original model.

**Figure 13:**
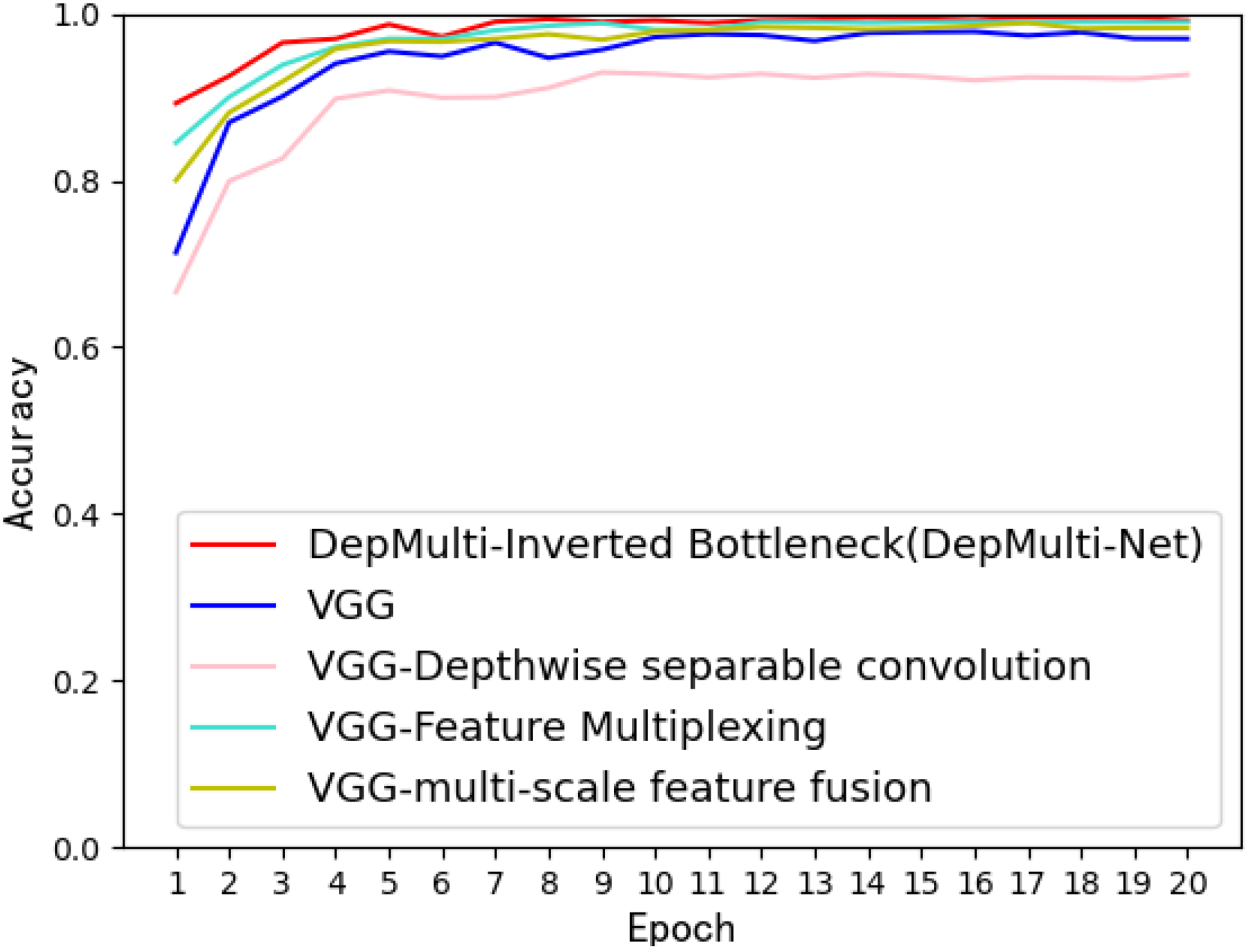
Comparison of recognition accuracy of each improved method.

Experimental results show that the use of depth - separable convolution, multi - scale feature fusion, feature reuse, and the inverted - bottleneck structure increased the recognition accuracy to 98.56%. The inverted - bottleneck structure can reduce the number of model parameters while balancing recognition accuracy. The comparison graph of recognition accuracy for each improved method is shown in **Figure 13**.

The performance metrics for each method comparison test are presented in **Table 7**. After incorporating depth - separable convolution, the multi - scale feature fusion module, the feature reuse module, and the inverted - bottleneck structure, the newly constructed DepMulti - Net model demonstrated the best performance in terms of recognition accuracy, precision, recall, and F1 score. Specifically, the precision, recall, and F1 score reached 97.06%, 97.64%, and 97.17%, respectively.

**Table 7:** Accuracy precision recall F1-score of different approaches.

Model	Accuracy rate	Precision	Recall	F1-score
VGG-16	95.79%	93.07%	92.51%	92.29%
VGG- Depthwise separable convolution	92.77%	90.42%	91.36%	90.89%
VGG- multi-scale feature fusion	96.41%	94.89%	94.06%	94.45%
VGG-Feature Multiplexing	97.53%	96.58%	95.89%	96.23%
VGG-Inverted Bottleneck(DepMulti-Net)	98.56%	97.06%	97.64%	97.17%

### 3.5 Comparison experiment of the DepMulti-Net and other models

To verify the overall performance of the model, the DepMulti - Net model based on the improved VGG was compared with common convolutional neural networks such as AlexNet, VGG - 16, ResNet - 18, DenseNet - 121, MobileNetV2, and ShuffleNetV2. Comparison experiments were conducted on the same dataset. The specific experimental data are shown in **Tables 8**, **9**. The results indicated that the DepMulti - Net model proposed in this study achieved an accuracy of 98.56% with a recognition time of only 0.073 seconds for a single disease image, while the parameter count was 13.50 million.

**Table 8:** Comparison test results of different models.

Model	Parameters/million	Accuracy	Rate
AlexNet	60M	95.07%	0.168S
VGG-16	138M	95.79%	0.296S
ResNet-18	25.5M	96.25%	0.101s
DenseNet-121	7.1M	96.53%	0.034S
MobileNetV3	4.2N	97.45%	0.021s
ShuffleNetV1	2.48M	96.28%	0.018s
DepMulti-Net	13.5M	98.56%	0.073S

**Table 9:** Comparison test results of different models.

Model	Bacteriablight	Blast	Brownspot	Tungro
AlexNet	95.17%	94.93%	95.28%	95.05%
VGG-16	97.00%	95.33%	95.00%	94.80%
ResNet-18	95.00%	97.08%	96.11%	96.00%
DenseNet-121	96.50%	96.53%	96.05%	97.23%
MobileNetV3	97.69%	97.19%	97.40%	97.68%
ShuffleNetV1	95.20%	97.20%	96.25%	96.64%
DepMulti-Net	98.625%	98.50%	98.56%	98.40%

The DepMulti - Net model outperformed common convolutional neural networks such as AlexNet, VGG - 16, ResNet - 18, and DenseNet - 121, as well as lightweight models such as MobileNetV2 and ShuffleNetV2. Specifically, AlexNet achieved an accuracy of 95.07% with 60 million parameters, VGG - 16 achieved 95.79% with 138 million parameters, and ResNet - 18 achieved 96.25% with 25.5 million parameters. DenseNet - 121 achieved an accuracy of 96.53% with 7.1 million parameters. It is evident that the DepMulti - Net model proposed in this study is superior to the aforementioned common convolutional neural networks.

**Figure 14:**
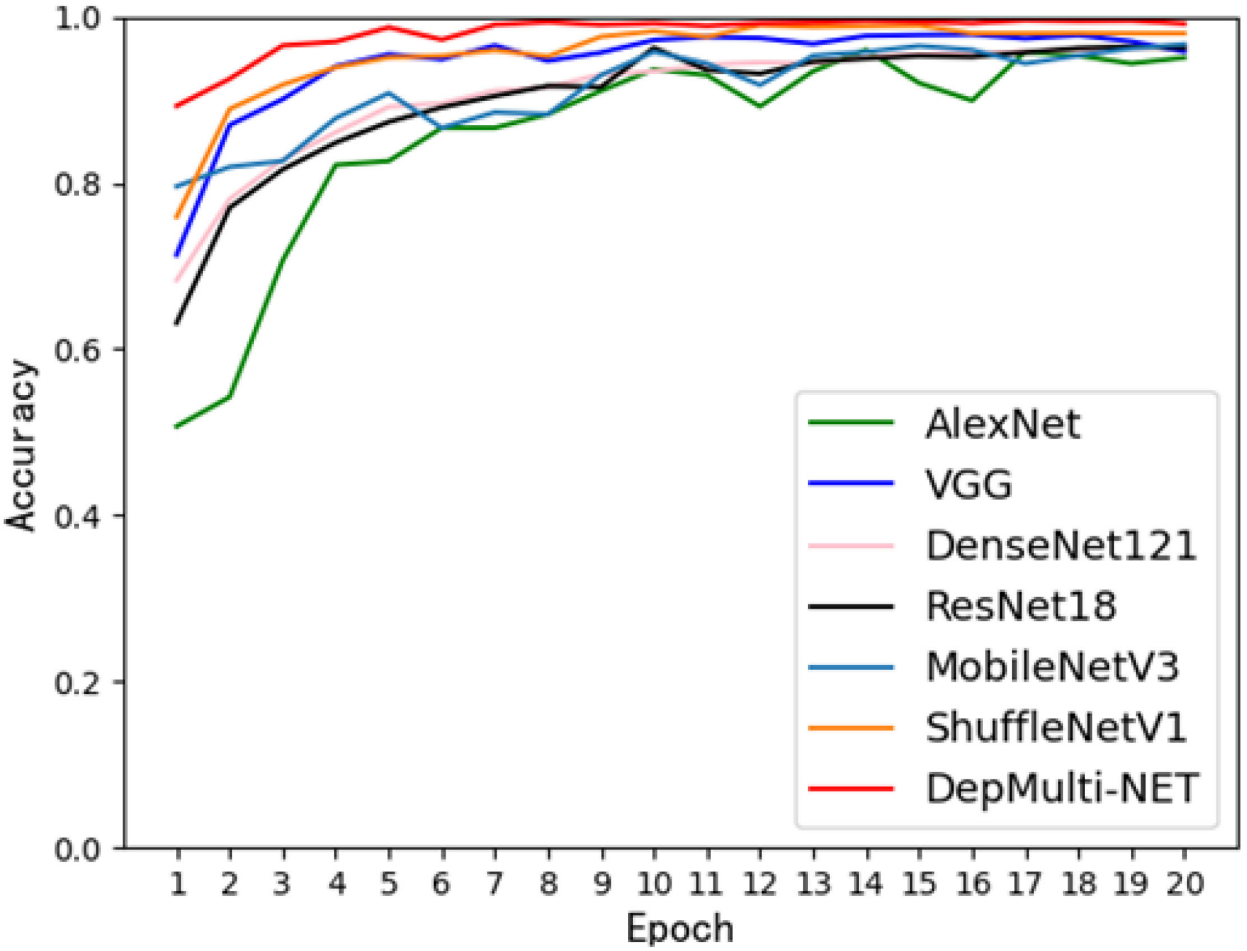
Comparison of recognition accuracy of each model.

Comparison tests were also conducted with lightweight models such as MobileNetV2 and ShuffleNetV2. Although these models have fewer parameters, their recognition accuracy was lower than that of the DepMulti - Net model in actual rice pest recognition tests. MobileNetV2 achieved a recognition accuracy of 97.45% with 4.2 million parameters, while ShuffleNetV2 achieved 96.28% with 2.48 million parameters. The combined comparison chart is shown in **Figure 14**.

The final experimental results demonstrate that the DepMulti - Net model based on the improved VGG outperforms common convolutional neural networks such as AlexNet, VGG - 16, ResNet - 18, and DenseNet - 121, as well as lightweight models such as MobileNetV2 and ShuffleNetV2, in terms of recognition accuracy. The model achieves high recognition accuracy with fewer parameters, effectively balancing the trade - off between model complexity and performance. This provides a valuable reference for addressing challenges such as limited crop pest datasets, excessive model parameters, and difficulties in model mobility and deployment.

### 3.6 Comparison between proposed method and empirical methods

The proposed method (DepMulti - Net) was compared with various empirical methods in image recognition tasks. The datasets involved included images of various plants, such as millet crop images, general plant images, ImageNet, NBAIR, and rice leaf images. The models used covered VGG - 16, VGGNet, ResNet - 101, AlexNet, GhostNet, RePVGG, Transformer, etc.

As shown in **Table 10**. In terms of accuracy, different research findings showed varying performance across respective datasets and models. For instance, Coulibaly et al. (2019) achieved an accuracy of 95.00% using VGG - 16 for millet crop images. Abdalla et al. (2019) obtained an accuracy of 93.00% with VGG - 16 for general plant images. On the ImageNet dataset, Chen et al. (2020) reached 91.83% with VGGNet, while Suh et al. (2018) achieved 98.00% using AlexNet. For the rice leaf image dataset, the accuracies of different models varied significantly. Zhou and Liu (2022) reported an accuracy of 79.38% with GhostNet, Gao et al. (2024) achieved 89.2% using RePVGG, Mannepalli et al. (2024) reached 97.77% with VGG - 16, and Amitabha Chakrabarty et al. (2024) obtained 97.00% with Transformer.

**Table 10:** Comparison between proposed method and empirical methods.

Authors	Dataset	Model	Accuracy
Coulibaly et al. (2019)	Millet crop images	VGG-16	95.00%
Abdalla et al. (2019)	Plant images	VGG-16	93.00%
Chen et al. (2020)	ImageNet	VGGNet	91.83%
Thenmozhi and Reddy (2019)	NBAIR	ResNet-101	95.02%
Suh et al. (2018)	ImageNet	AlexNet	98.00%
Mannepalli et al. (2024)	RiceLeaf	VGG-16	97.77%
Zhou and Liu (2022)	RiceLeaf	GhostNet	79. 38%
Gao et al. (2024)	RiceLeaf	RePVGG	89.2%
Chakrabarty et al. (2024)	RiceLeaf	Tansformer	97.00%
Proposed Method	RiceLeaf	DepMulti-Net	98.56%

The proposed method, using the DepMulti - Net model, achieved an accuracy of 98.60% on the rice leaf image dataset, surpassing other empirical methods. This indicates that the proposed method has a significant advantage in rice leaf image recognition tasks, with outstanding performance. It holds potential for application and research value in the field of plant image recognition.

### 3.7 The differences between this study and other leaf disease recognition studies

This study introduces a novel leaf disease identification model (DepMulti-Net) by incorporating depthwise separable convolution and multi-scale feature fusion techniques, significantly enhancing the model’s recognition accuracy and lightweight nature. As shown in **Table 11**. Compared to existing research, the main differences and innovations of this study are as follows.

**Table 11:** Comparison of leaf disease identification studies.

Feature	Our Study(DepMulti-Net)	Existing Research
Dataset	Real paddy field environmental data, complex backgrounds	Laboratory environmental data, single background
Feature Extraction	Multi-scale feature fusion, suitable for different scale lesions	Single-scale feature extraction, limited capability for small lesion recognition
Recognition Accuracy	98.56%, excellent performance in complex backgrounds	Poor adaptability in complex backgrounds
Model Lightweight	Parameter count is only 13.5M, suitable for edge device deployment	Larger parameter count
Practical Application	Suitable for drones, mobile phones, and other lightweight platforms	Mainly for high-performance computing platforms

The DepMulti-Net model proposed in this study demonstrates outstanding performance in rice leaf disease identification tasks. Compared to existing research, this study utilizes a dataset from real paddy fields with complex backgrounds, whereas current studies often employ datasets from laboratory environments with more uniform backgrounds. The feature extraction method in this study integrates multi-scale features, making it suitable for lesions of varying sizes, while existing research, which typically extracts features at a single scale, has limited capability in recognizing small disease spots.

In terms of recognition accuracy, DepMulti-Net achieves an impressive 98.56% accuracy rate, significantly outperforming existing methods that show poor adaptability in complex backgrounds. The model is also lightweight, with a parameter count of only 13.5 million, making it highly suitable for deployment on edge devices such as drones and mobile phones. This contrasts with existing research that generally requires high-performance computing platforms due to larger parameter counts.

Overall, DepMulti-Net offers a practical and efficient solution for leaf disease identification, with a strong emphasis on real-world applicability and high accuracy in complex environments.

## 4 Discussion

The DepMulti-Net model proposed in this study has demonstrated exceptional performance in rice disease identification tasks, achieving an average recognition accuracy of 98.56% with only 13.50 million parameters. This result significantly outperforms various existing convolutional neural networks and lightweight models. To comprehensively evaluate the contributions of this study, we compared its results with those of similar research and further explored the model’s strengths, limitations, and potential directions for future improvement.

In existing research on rice disease identification, many scholars have employed different deep - learning models and methods. For instance, Chen et al. utilized a pre - trained VGG network on the ImageNet dataset for transfer learning, achieving an average recognition accuracy of 92.00% (Chen et al., 2020). However, their model’s large parameter size made real - time recognition challenging. In contrast, the DepMulti - Net model proposed in this study significantly reduced the number of parameters while improving recognition accuracy by 6.56 percentage points, making it more suitable for practical applications in agricultural environments. Mannepalli et al. proposed a rice disease identification method based on the VGG16 network, achieving an accuracy of 97.77% (Mannepalli et al., 2024). However, their model’s parameter size was as high as 138 million, far exceeding that of the DepMulti - Net model proposed in this study. Although their accuracy was high, the large parameter size limited its deployment on mobile or edge computing devices. By employing depthwise separable convolution and multi - scale feature fusion techniques, this study maintained high accuracy while significantly reducing the model’s parameter size, offering greater flexibility for practical applications. Additionally, Zhou et al. proposed a lightweight rice disease identification method based on YOLOv4 - GhostNet, but their average accuracy was only 79.38%, with a parameter size of 42.45 million (Zhou and Liu, 2022). In comparison, DepMulti - Net achieved a nearly 20 percentage point improvement in accuracy with only 13.50 million parameters, demonstrating its superior balance between lightweight design and high accuracy.

The DepMulti - Net model proposed in this study exhibits significant advantages in the following aspects:

Through depthwise separable convolution and feature reuse techniques, the model’s parameter size was significantly reduced to only 9.7% of that of VGG - 16. This makes it suitable for deployment on resource - constrained devices such as mobile devices or drones. Supported by multi - scale feature fusion and inverted bottleneck structures, the model’s ability to extract disease features in complex backgrounds was significantly enhanced. It achieved a recognition accuracy of 98.56%, outperforming most existing models. Through data augmentation techniques and the construction of a dataset from real - field environments, the model demonstrated stable performance under complex backgrounds and varying lighting conditions, exhibiting strong generalization capabilities.

Despite the DepMulti - Net model’s excellent performance in rice disease identification, it still has some limitations:

This study focused on only four common rice diseases. For rare disease categories, the model’s recognition capabilities may be insufficient. Future work should expand the dataset to include more disease types to improve the model’s generalizability. In the multi - scale feature fusion module, the choice of convolutional kernels significantly impacts recognition results. Although this study selected 1×1, 3×3, and 5×5 convolutional kernels through experiments, further optimization of kernel sizes and combinations may be necessary for different disease scales. The DepMulti - Net model was primarily designed for rice disease identification, and its performance in identifying diseases in other crops has not been validated. Future research could explore the application of this model to disease identification tasks for other crops and further optimize the model structure to adapt to the characteristics of different crops.

Based on the achievements and limitations of this study, future research directions may include the following:

Collecting more types of rice disease images, especially those of rare diseases, to enhance the model’s generalization ability. Additionally, introducing disease datasets for other crops could explore the model’s potential in cross - crop disease identification. Further experiments and statistical analyses could optimize the sizes and combinations of convolutional kernels in the multi - scale feature fusion module to improve the model’s ability to recognize disease features at different scales. Although DepMulti - Net has achieved a high level of lightweight design, further exploration of model compression and acceleration techniques, such as quantization and pruning, could enhance the model’s real - time performance and deployment efficiency. Investigating how to apply the DepMulti - Net model to disease identification tasks for other crops and exploring a universal model for crop disease identification could provide broader support for smart agriculture.

## 5 Conclusion

This study focuses on identifying four common rice leaf diseases: Blast, Brownspot, Tungro, and Bacterial blight. By integrating depthwise separable convolution, feature reuse techniques, and inverted bottleneck structures, we developed a feature reuse and multi - scale feature fusion - based inverted bottleneck module, proposing the DepMulti - Net model. Comparative experiments with various convolutional neural networks yielded the following key conclusions:

1. The proposed DepMulti - Net model achieved an average recognition accuracy of 98.56% with only 13.50 million parameters, significantly outperforming existing rice disease identification methods. The use of depthwise separable convolution substantially reduced the number of parameters, addressing the issues of high parameter counts, extensive training data requirements, and difficulties in real - time recognition associated with traditional deep - learning models. This provides an efficient and lightweight solution for crop disease identification.
2. To address the challenges of complex background interference and difficulty in disease feature extraction in rice disease identification, this study introduced a multi - scale feature fusion module and a feature reuse mechanism. These enhancements effectively improved the model’s ability to extract disease features in complex backgrounds, particularly excelling in identifying lesions of varying scales. The incorporation of the inverted bottleneck structure further enhanced the model’s precision in recognizing fine - grained disease features, ensuring its applicability in real - world field environments.
3. This study constructed a rice disease dataset comprising 20,000 images, covering the four common rice diseases, and enhanced data diversity through data augmentation techniques. This dataset serves as a valuable resource for future research, advancing the field of rice disease identification.
4. Through comparative experiments with widely used convolutional neural networks and lightweight models such as AlexNet, VGG - 16, ResNet - 18, DenseNet - 121, MobileNetV3, and ShuffleNetV1, DepMulti - Net achieved an optimal balance between parameter count and recognition accuracy, significantly outperforming existing models. The model is not only suitable for rice disease identification but also provides a lightweight solution that can be adapted for other crop disease identification tasks.

The practical application value of this study lies in providing an efficient and lightweight disease identification tool for smart agriculture, enabling farmers to promptly identify and control rice diseases, thereby reducing economic losses and ensuring food security. Future work will focus on expanding the dataset to include more disease categories and images under various environmental conditions to enhance the model’s generalization capabilities. Additionally, we will explore the potential of applying this model to the identification of diseases in other crops.

## Data Availability

The original contributions presented in the study are included in the article/supplementary material. Further inquiries can be directed to the corresponding author.

## Appendix A

Dataset and code are available at: https://github.com/kuihu-hk.
